# Different Auditory Feedback Control for Echolocation and Communication in Horseshoe Bats

**DOI:** 10.1371/journal.pone.0062710

**Published:** 2013-04-24

**Authors:** Ying Liu, Jiang Feng, Walter Metzner

**Affiliations:** 1 Jilin Key Laboratory of Animal Resource Conservation and Utilization, Northeast Normal University, Changchun, Jilin, China; 2 Department Integrative Biology and Physiology, University of California Los Angeles, Los Angeles, California, United States of America; 3 Neurosensing and Bionavigation Research Center, Doshisha University, Kyotanabe, Kyoto, Japan; University of Lethbridge, Canada

## Abstract

Auditory feedback from the animal's own voice is essential during bat echolocation: to optimize signal detection, bats continuously adjust various call parameters in response to changing echo signals. Auditory feedback seems also necessary for controlling many bat communication calls, although it remains unclear how auditory feedback control differs in echolocation and communication. We tackled this question by analyzing echolocation and communication in greater horseshoe bats, whose echolocation pulses are dominated by a constant frequency component that matches the frequency range they hear best. To maintain echoes within this “auditory fovea”, horseshoe bats constantly adjust their echolocation call frequency depending on the frequency of the returning echo signal. This Doppler-shift compensation (DSC) behavior represents one of the most precise forms of sensory-motor feedback known. We examined the variability of echolocation pulses emitted at rest (resting frequencies, RFs) and one type of communication signal which resembles an echolocation pulse but is much shorter (short constant frequency communication calls, SCFs) and produced only during social interactions. We found that while RFs varied from day to day, corroborating earlier studies in other constant frequency bats, SCF-frequencies remained unchanged. In addition, RFs overlapped for some bats whereas SCF-frequencies were always distinctly different. This indicates that auditory feedback during echolocation changed with varying RFs but remained constant or may have been absent during emission of SCF calls for communication. This fundamentally different feedback mechanism for echolocation and communication may have enabled these bats to use SCF calls for individual recognition whereas they adjusted RF calls to accommodate the daily shifts of their auditory fovea.

## Introduction

Hearing one's own voice is critical for the maintenance of stable vocalizations in humans and songbirds, even in adulthood after human speech and bird song have already been established (for review [1],[2]). While in most other mammals, such as non-human primates, rodents or cats, such auditory feedback appears to play a somewhat minor role (reviewed in [2], [3]), it is quintessential in echolocating bats: they continuously adjust the spectrotemporal features of their sonar pulses in order to optimize the detection of the corresponding echo signals. In addition to producing echolocation pulses, bats generate a large repertoire of social calls to communicate with one another [4]–[14]. It appears that at least some of these communication signals are also dependent on auditory feedback, as for example effects of vocal learning suggest [2], [15]–[17]. Not only do bats modify the fine structure of their vocalizations based on social experience, they can even acquire new vocalizations through vocal imitation [17].

Interestingly, the neuronal premotor networks underlying the two forms of vocalization appear to differ somewhat within the bat's brain. Some midbrain structures control only echolocation pulses and others only communication signals [18]. In mammals in general, different types of vocalization appear to involve different subsystems of the brainstem vocal motor network [19]. It is unclear, however, if the different vocal premotor networks also receive different auditory feedback. Here we examined the role of auditory feedback for echolocation pulses emitted at rest (resting frequency, RF), i.e. when stationary and not flying, and one particular type of communication signals in greater horseshoe bats, *Rhinolophus ferrumequinum*. Greater horseshoe bats emit echolocation calls that are dominated by a constant frequency (CF) component and have durations between 30 and 50 ms [20]–[22]. The communication call we focused on here resembles an echolocation pulse but is much shorter (around 17 ms; SCFs) and produced only during social interactions [11].

“Constant-frequency-bats”, such as horseshoe bats or the neotropical mustached bat, *Pteronotus parnellii*, are highly specialized insect hunters. They forage close to or even within dense vegetation and thus have to deal with heavy echo cluttering caused by reflections from the background vegetation (e.g., [21], [23], [24]). The long, narrow-band echolocation signals enable them to utilize echo cues that are caused by the wing beats of flying insect which they prey upon. Fluttering insects cause frequency modulations in the returning echoes, so called “acoustic glints” [25], that contain the information necessary for the bat to detect and recognize its prey (e.g., [24], [26]).

The basis for this remarkable echolocation ability is provided by specializations within the auditory system of these bats. A filter mechanism that is narrowly tuned to the echo's narrowband frequency component rejects background clutter while it helps to detect acoustic glints. This auditory filter is found in the cochlea and termed an “auditory fovea” [24], [27]–[30].

Horseshoe and mustached bats inevitably face one particular problem by using such narrowband echolocation signals: During flight, the movement of the bat relative to the background, such as dense vegetation, induces additional Doppler-shifts in the echo frequency. In contrast to Doppler-effects caused by fluttering insects, the bat's flight induces shifts of the entire echo signal away from the frequency that is emitted in resting bats, i.e. the “resting frequency” (RF). As a consequence, echoes drop out of the auditory fovea resulting in loss of vital echo information. To compensate for these flight-induced Doppler-effects in the returning echo, CF- bats continuously adjust the frequency of their echolocation calls (Doppler-shift compensation behavior, DSC [22], [31], [32]. Control of echolocation call frequencies during flight (DSC) and at rest (resting frequencies) represents one of the most precise forms of sensory-motor integration known [33], [34].

Interestingly, RFs emitted by horseshoe bats and other constant frequency bats do not remain constant but instead change over time [35]–[39]. Daily variations of more than 1 kHz, which were not associated with DSC, have been reported in the Taiwanese leaf-nosed bat, *Hipposideros terasensis* [35], and in the mustached bat, RFs changed with changes in body temperature by approximately 100 Hz/°C ([36], [37]; see also Discussion).

In addition to emitting calls for echolocation, bats also generate sounds for communication. Most communication signals are spectrotemporally more complex than sonar pulses [e.g., [7], ]. The behavioral context served by communication calls includes courtship and mating behavior, foraging, group bonding, distress, and reunion between offspring and mother, and can vary from species to species [5], [6], [8], [14], [15], [40]–[42]. In the Mexican free-tailed bat, it has been shown that communication calls are distinct across individuals [43]. Therefore they may carry some “individual signature” to signal the audience who is calling. In order to maintain individuality, it would therefore be beneficial to maintain a distinct communication call type or pattern to facilitate individual recognition by others. It should be noted that echolocation calls can also carry individual characters and thus be used to recognize individuals or facilitate the detection of echoes in areas with vocalizing conspecifics [21], [44]–[46]. We tested this idea of emitting distinct communication signals by analyzing the variability of a particularly simple type of communication call in greater horseshoe bats, the short constant frequency call (SCF; [11]). SCFs resemble echolocation pulses but are much shorter and produced only in social contexts. Although their precise behavioral meaning is still unknown, male horseshoe bats emit SCFs during mating, or either sex can produce SCFs during interactions reminiscent of “greeting behavior” when two bats briefly and softly tap each other with their wings [11]. SCFs represent a distinct call type that is different from echolocation calls [11]: when analyzing the duration of all horseshoe bat calls that were dominated by a constant frequency component, including echolocation calls, Ma et al. (2006) found three clearly normally distributed populations of call durations (Kolmogorov-Smirnov Test). SCF calls had average durations of 14.7±3.53 ms (range: 11.3 to 32.0 ms), the intermediate or “normal” constant frequency (CF) calls (corresponding to normal echolocation pulses) had a mean duration of 54.0±17.5 ms (range: 22 to 126 ms), and long constant frequency (LCF) calls were longer than 132 ms. The distribution for SCF syllables and ”normal” constant frequency (echolocation) calls overlapped between 22 ms and 32 ms and it was statistically not possible to assign calls within this range to only SCF or “normal” constant frequency (echolocation) calls. Therefore, SCF calls were conservatively defined as those with durations of 22 ms or shorter, and ”normal” constant frequency (echolocation) calls as those ranging from 24 to 126 ms.

In the present study, we found that whereas “normal” constant frequency (echolocation) calls emitted at rest (resting frequencies, RFs) varied from day to day by several hundred Hz, SCF values varied considerably less. As a result, RFs overlapped between many of the individuals but SCFs remained significantly different for most bats. We suggest that this may indicate a significant difference in how the motor control of RFs and SFCs, respectively, depends on auditory feedback.

## Results

We recorded a total of 1754 RF and 4275 SCF calls from 8 greater horseshoe bats (Table 1). As in a previous characterization of the repertoire of social calls in horseshoe bats [11], we found that SCFs had on average less than half the duration of RF calls. In our sample, the average duration of RF calls was 51.7±12.81 ms (Kolmogorov-Smirnov test *P* = 0.067) and that of SCFs 15.4±3.59 ms (Kolmogorov-Smirnov test P = 0.128). Another feature that clearly helped in distinguishing SCF from RF calls was that horseshoe bats switched between the two call types very abruptly. We never observed a gradual transition between normal echolocation pulses and SCF calls as would be expected if SCFs were part of an approach phase during normal echolocation behavior [21], [47]. SCF calls were also never emitted as solitary calls but instead were always part of a longer sequence (see also [11]). The number of SCFs within a sequence varied between 2 and 17, with a typical average between 5 to 6 calls per sequence (Table 1). There also was a large inter-individual variation in the number of SCF sequences produced by each bat, ranging from under 20 to more than 300 (total number for all bats: 766; Table 1), and thus the total number of SCF calls, ranging from less than 70 to more than 2000 (Table 1).

**Table 1:** Summary of data samples used for analysis: number of days on which bats produced SCF and RF calls (expressed as total numbers), and number of SCF calls produced in each sequence. m: male, f: female.

Individual	Number of days	Total number of RF calls	Total number of SCF calls	Total number of SCF sequences	Number of SCFs per sequence: range/mean ± SD
1m	2	91	159	25	3–12/6.36±2.45
2m	4	281	2009	315	3–17/6.39±3.91
3f	3	306	560	110	2–9/5.04±2.53
4f	2	61	67	18	2–9/3.72±2.47
5m	2	314	273	47	3–13/5.81±2.44
6m	2	83	196	26	2–13/5.30±2.89
7m	4	315	843	192	2–13/4.32±2.19
8m	2	303	168	33	3–11/5.26±2.14

**Figure 1:**
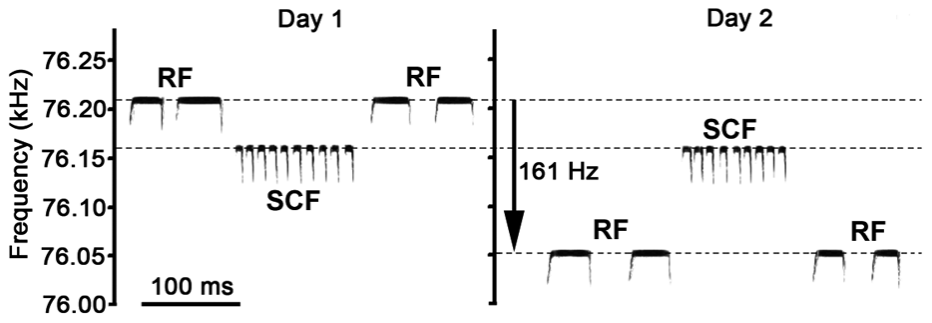
Sonagram of echolocation pulses emitted at rest (RF) and short CF (SCF) communication calls of one individual bat (male 6m) on two different days. In each panel (“Day 1” and ”Day 2”), RFs are given before and after social interactions with a female bat, reminiscent of “greeting behavior”, during which the male produced SCFs. The 3 call sequences given for each day were each approximately 30 min apart.

The mean frequency of all RFs by all bats combined and averaged over the entire observation period was 76.67±0.102 kHz and those of SCFs 76.68±0.053 kHz. When analyzing the daily variation of call frequencies, we found that RFs varied greatly from day to day, sometimes by more than 900 Hz, whereas SCFs remained extremely constant. Figure 1 exemplifies these differences for calls emitted by the same bat (male bat 6 m) before, during, and after “greeting-like” interactions with a female bat, in which the male's RF within 10 s before and after these interactions was 76.215±0.03 kHz (n = 26) on the first and 76.054±0.03 kHz (n = 20) on the subsequent day. SCFs, however, remained at 76.163±0.02 kHz (n = 19 each day).

Such greater day-to-day variations in RFs than in SCFs were a common feature in all 8 bats analyzed and for all days compared. Table 2 (left half) lists the variations in peak frequencies for RF and SCF calls for each individual bat and different day-to-day comparisons. It shows that the RFs emitted on different days varied much more than the frequencies of SCF calls in all bats and all day-to-day comparisons. The significance levels (*P_day-to-day_* values in Table 2) were all >>0.1 (average 0.281) for SCF comparisons whereas they were <0.01 when comparing RFs on different days. Table 2 also shows that the peak frequencies of RF calls emitted immediately before a SCF sequence did not differ significantly from those emitted immediately afterwards: the significance levels (P_RF_ values in Table 2) were in most cases >>0.1 (average 0.502). (The duration values for RF and SCF calls that are presented in the right half of Table 2 are compared at the end of the Results).

**Table 2:** Day-to-day comparisons of peak frequencies (left half) and durations (right half) of RF and SCF calls produced by all 8 individuals.

Bat	Observation days	Peak frequency (kHz)				Duration (ms)			
		SCF	RF before SCF	RF after SCF	*P*_RF_	SCF	RF before SCF	RF after SCF	*P*_D_
1m	Day1	77.43±0.03	77.45±0.13	77.48±0.05	0.213	17.85±4.36	52.74±13.60	49.27±7.49	0.427
	Day2	77.46±0.03	77.39±0.04	77.37±0.04	0.581	15.70±3.61	36.88±10.98	35.71±4.33	0.851
	*P_day-to-day_*	0.206	<0.01	<0.01		0.174	<0.01	0.003	
2m	Day1	77.31±0.04	77.33±0.06	77.34±0.03	0.464	16.48±4.80	48.85±12.64	51.45±22.54	<0.01
	Day2	77.30±0.04	77.23±0.03	77.25±0.04	0.402	15.79±2.60	53.77±17.14	53.46±13.95	0.538
	Day3	77.32±0.05	77.47±0.04	77.46±0.06	0.253	19.56±5.73	42.35±9.13	42.53±10.62	0.974
	Day4	77.31±0.04	77.48±0.05	77.48±0.07	0.960	17.48±5.62	60.73±16.37	54.95±15.75	<0.01
	*P_day-to-day_*	0.369	<0.01	<0.01		<0.01	<0.01	<0.01	
3f	Day1	76.96±0.04	76.86±0.05	76.87±0.04	0.212	17.58±3.72	40.51±13.59	38.58±6.90	<0.01
	Day2	76.99±0.05	77.02±0.05	77.04±0.06	0.226	16.84±2.01	26.37±6.45	36.17±8.82	<0.01
	Day3	76.96±0.03	76.96±0.04	76.96±0.03	0.157	20.04±2.48	28.22±6.47	29.04±4.96	0.11
	*P_day-to-day_*	0.352	<0.01	<0.01		<0.01	<0.01	<0.01	
4f	Day1	76.83±0.04	76.75±0.04	76.79±0.05	0.104	17.42±2.47	39.88±11.97	34.33±7.40	0.204
	Day2	76.87±0.08	76.92±0.08	76.93±0.09	0.889	15.47±2.64	31.83±4.95	35.61±11.32	0.516
	*P_day-to-day_*	0.296	<0.01	<0.01		0.109	<0.01	0.438	
5m	Day1	76.67±0.06	76.93±0.08	76.92±0.08	0.418	18.20±2.49	33.52±5.37	48.21±13.95	0.001
	Day2	76.66±0.06	76.74±0.04	76.75±0.04	0.417	12.97±1.67	43.60±13.92	58.74±18.38	0.083
	*P_day-to-day_*	0.572	<0.01	<0.01		<0.001	<0.01	<0.01	
6m	Day1	76.20±0.06	76.17±0.06	76.15±0.06	0.495	11.97±2.97	28.23±5.12	26.45±4.42	0.610
	Day2	76.16±0.05	75.91±0.11	75.91±0.10	0.856	10.38±2.35	29.77±6.56	30.55±6.31	0.772
	*P_day-to-day_*	0.114	<0.01	<0.01		0.344	0.595	<0.01	
7m	Day1	76.00±0.05	76.01±0.04	76.01±0.03	0.760	12.97±1.67	33.78±7.91	30.41±9.08	0.540
	Day2	76.02±0.04	75.94±0.06	75.94±0.05	0.748	16.35±2.73	37.19±5.81	44.01±12.30	0.148
	Day3	76.01±0.04	75.87±0.06	75.87±0.03	0.777	17.83±3.52	48.52±11.48	32.3±5.25	<0.01
	Day4	76.01±0.04	75.79±0.05	75.80±0.07	0.661	16.62±3.39	34.53±10.23	37.23±4.56	0.017
	*P_day-to-day_*	0.130	<0.01	<0.01		<0.01	<0.01	<0.01	
8m	Day1	76.04±0.09	76.03±0.04	76.03±0.04	0.808	15.97±3.25	41.16±9.85	38.39±8.39	0.167
	Day2	76.01±0.04	75.93±0.04	75.94±0.03	0.137	18.85±3.31	40.58±8.41	47.08±14.75	0.005
	*P_day-to-day_*	0.214	<0.01	<0.01		0.035	0.001	<0.01	

Frequency and duration values for RF calls are given as averages for 10 calls emitted immediately before and after a sequence of SCF calls. The number of SCF syllables analyzed is given in Table 1. *P_day-to-day_*: significance levels (ANOVA) for comparing RF and SCF calls emitted on different days. *P*_RF_: significance levels for comparing peak frequencies of RF calls emitted immediately before and after a SCF sequence. *P_D_*: significance levels for comparing durations of RF calls emitted immediately before and after a SCF sequence.

**Figure 2:**
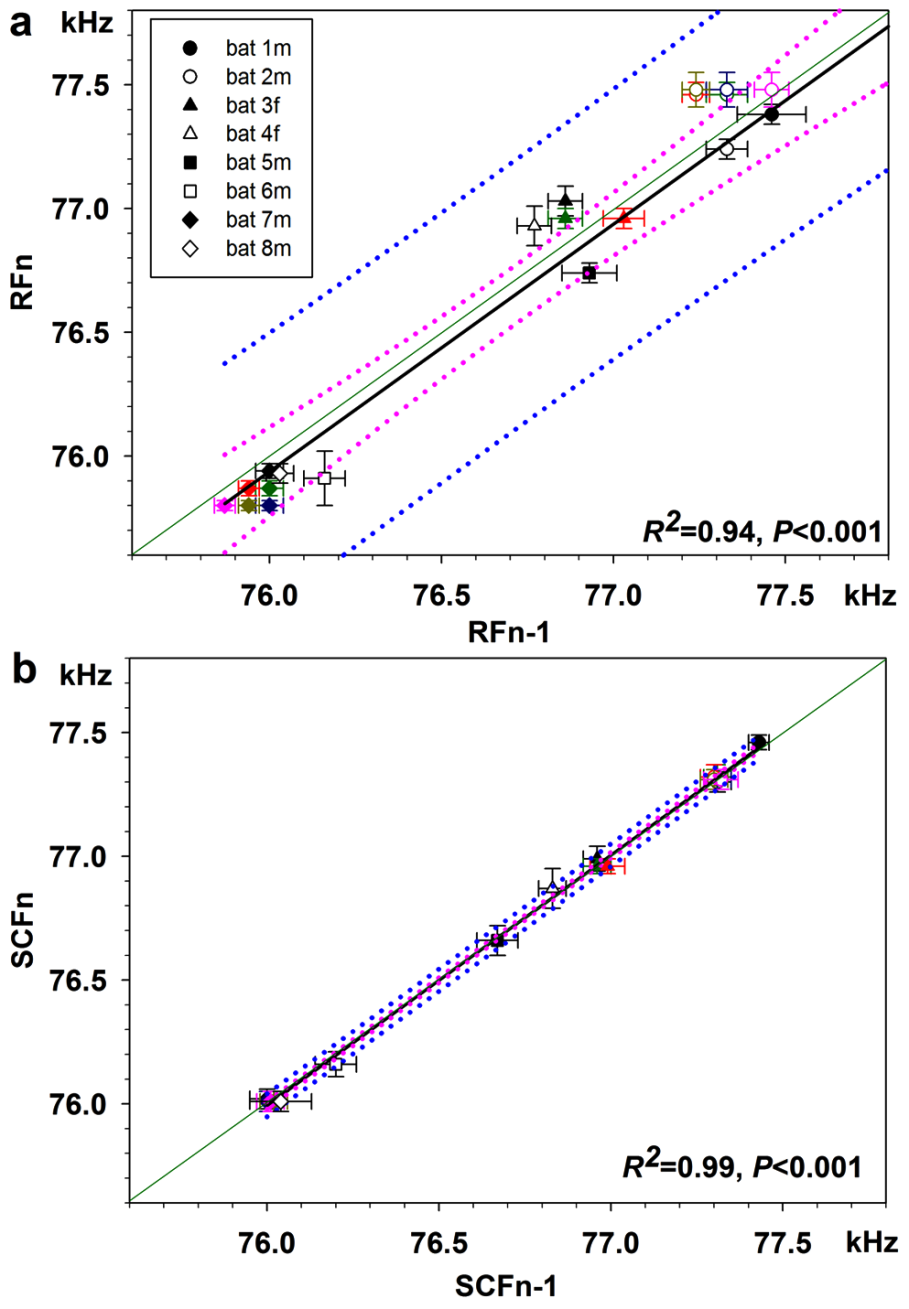
Comparison of peak frequencies of RF and SCF calls for each bat (indicated by different symbols, see a.) and for all days tested (indicated by different colors as noted below). Same data as in Table 2 (left half) with peak frequencies for RF calls emitted before and after a SCF sequence being averaged. Solid black lines indicate the regression line with *R^2^* and *P*-values given in each subfigure, dotted pink lines give the 95% confidence intervals, and dotted blue lines outline the predicted intervals. a. RFs on one sample day (RF_n_) compared with RFs of a previous day (RF_n−1_) (black: day 2 vs. day 1, dark green: day 3 vs. day 1, dark blue: day 4 vs. day 1, red: day 3 vs. day 2, dark yellow: day 4 vs. day 2, pink: day 4 vs. day 3). b. SCFs on one sample day (SCF_n_) compared with SCFs on a previous day (SCF_n−1_) (same color scheme as in a.).

Figure 2 illustrates the daily variations in the peak frequencies of RF and SCF calls graphically. RFs emitted on one day (RF_n_) relative to those emitted on the previous day (RF_n−1_) (Fig. 2a) clearly exhibited a greater variability that SCFs emitted by the same bats on the same days (Fig. 2b): the standard deviations (error bars), 95% confidence intervals (dotted pink lines), and predicted intervals (dotted blue lines) were all greater for RFs (Fig. 2a) than for SCFs (Fig. 2b).

**Figure 3:**
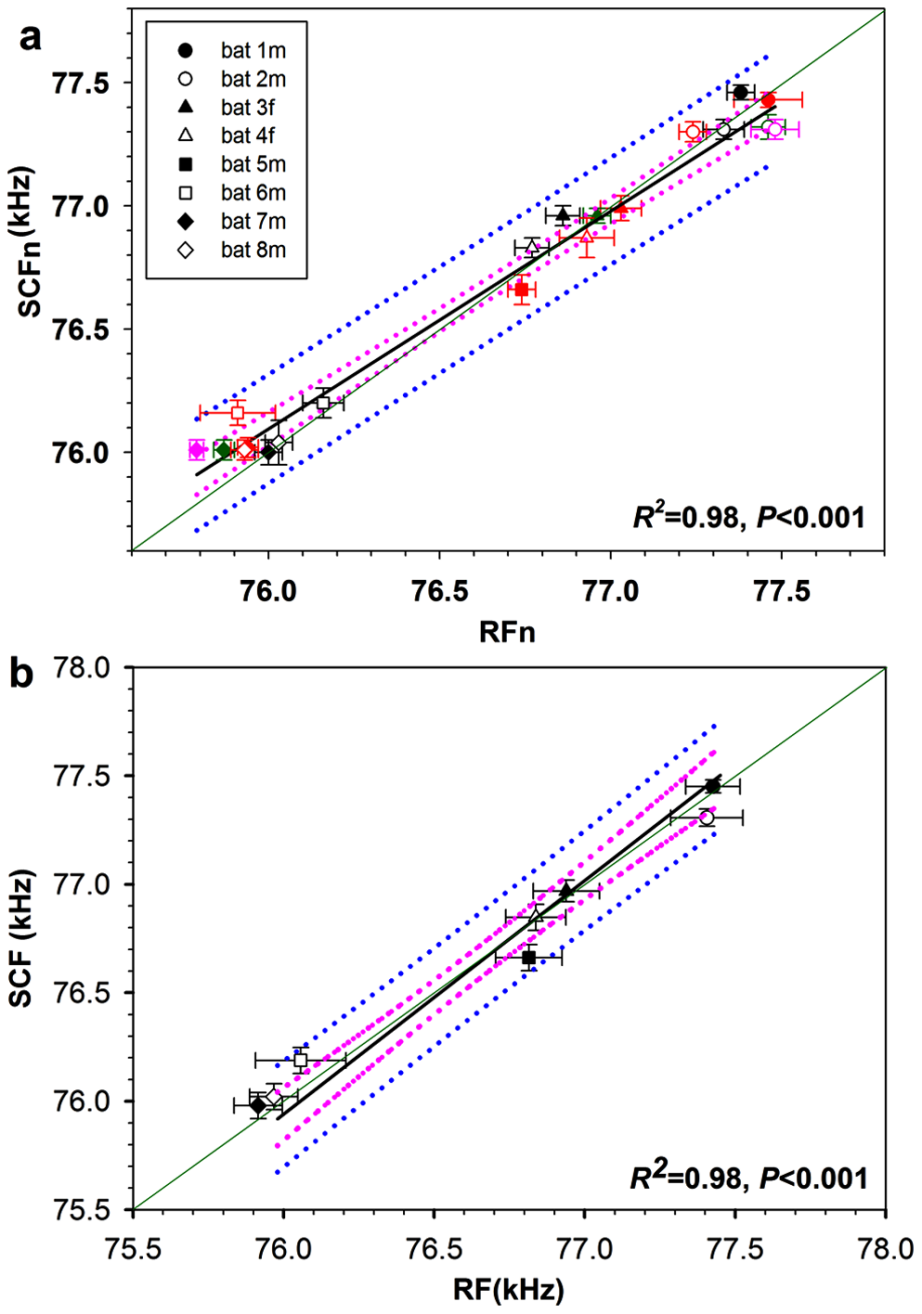
Distribution of RFs and SCF frequencies for all 8 bats. Solid black lines indicate the regression line with *R^2^* and *P*-values given in each subfigure, dotted pink lines give the 95% confidence intervals, and dotted blue lines outline the predicted intervals. a. individual RFs compared with SCFs for each bat and each day (black: day 1 vs. day 1, red: day 2 vs. day 2, dark green: day 3 vs. day 3, pink: day 4 vs. day 4). b. same data as in a. but averaged over the entire observation period and presented as mean ± SD.

Accordingly, when we compared the variation in the peak frequencies of RF calls with that of SCF calls (Figure 3), we found that in most cases RFs overlapped between different days and individuals whereas SCFs did less so (Fig. 3a). This becomes more evident when plotting the average peak frequencies for RF and SCF calls and their variability for all 8 individuals (Fig. 3b). As for the individual data (Fig. 3a), the averaged data yielded 3 clusters, most likely due to our somewhat limited sample size of 8 bats. Except for 2 individuals (bats 3f in the center cluster and 6 m in the left cluster), the RFs within each cluster were not significantly different (Tables S1, S2; ANOVA, significance level 0.05). In contrast, SCF frequencies of all 8 bats did differ significantly from one another (Tables S1, S2; ANOVA, significance level 0.05). For all bats combined, the average variation in the peak frequencies of RF calls over the entire observation period was 693.8±352.98 Hz (n = 1754 calls, Table 1) whereas SCF peak frequencies varied by merely 245.0±78.19 Hz (n = 4275, Table 1), which is only 35% of the variation observed for RFs.

In contrast to peak frequencies, call durations always differed significantly between these days for both RF as well as SCF calls (Table 2, right half). On average, the durations of RF calls varied by 46.68±19.46 ms and those of SCFs by 14.28±5.99 ms, corresponding to 121% and 85% of their respective average durations.

**Figure 4:**
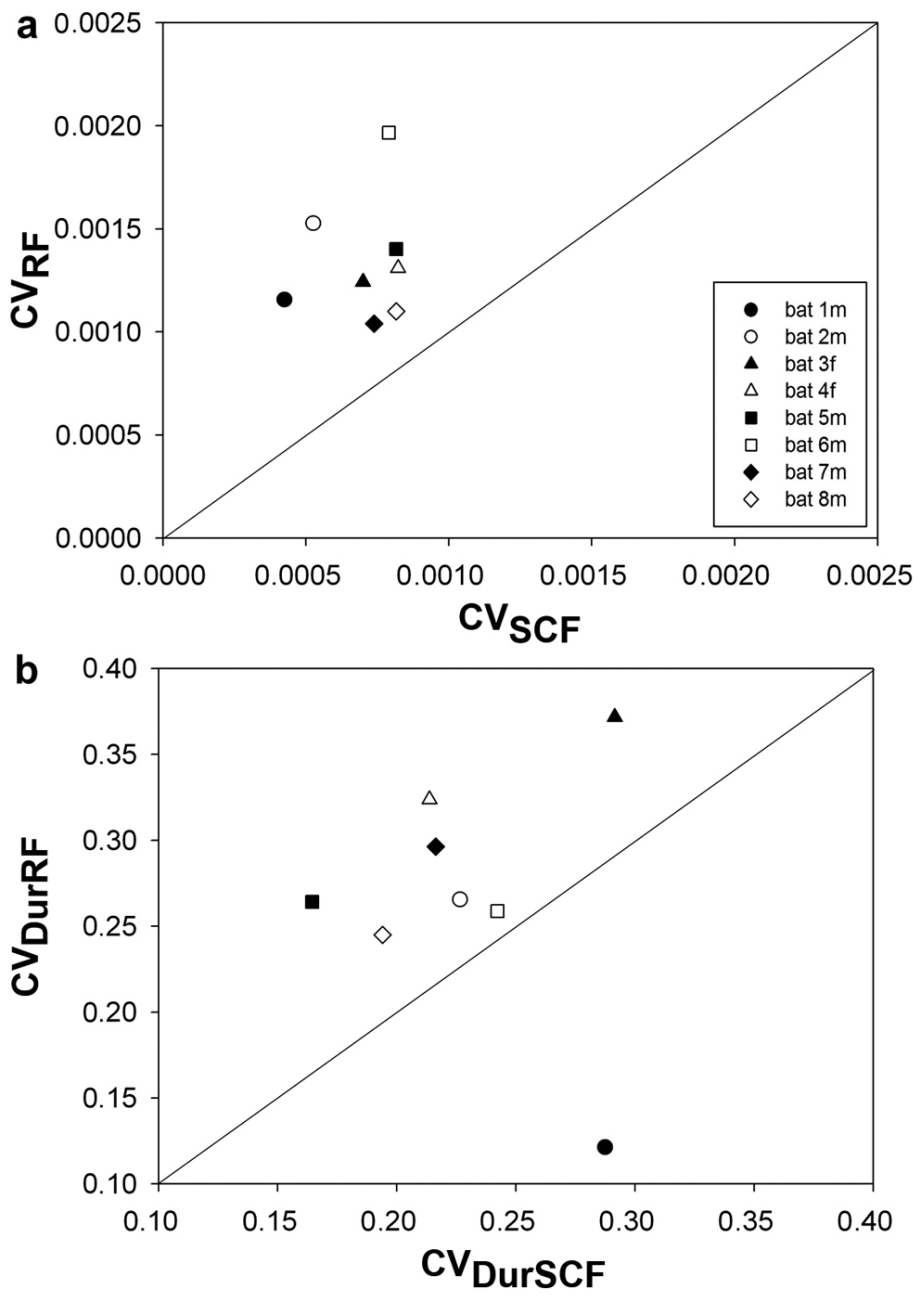
Comparison of CVs (SD/mean) for peak frequency values (a.) and durations (b) for all RF and SCF calls recorded from all bats over the entire observation period. Note the different scales for a. and b., respectively.

**Figure 5:**
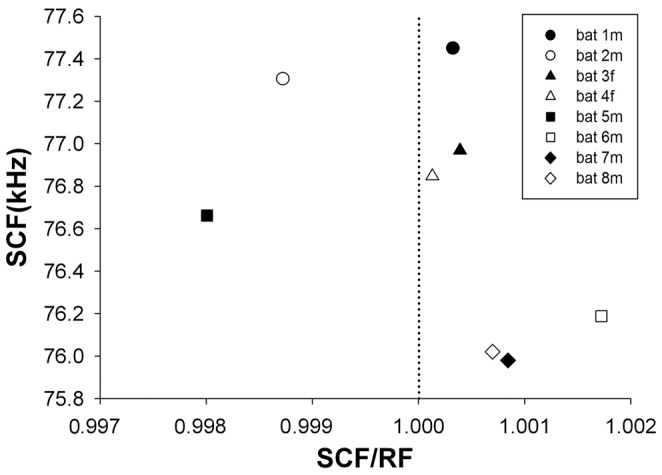
Distribution of SCF frequencies versus the ratio of SCF/RF for all individuals and over the entire observation period.

As a result of the larger variability of RFs, the CVs (mean over SD) for the peak frequencies of RF calls were approximately twice as large as those of SCF calls (Figure 4a; mean ± SD for RFs: 0.00134±0.000298, and for SCFs:. 0.00070±0.00015, independent samples, T-test, *t* = 5.411, *P* = 0.000). RFs for individual bats could switch between values above and below those for SCFs (Table 2) and SCFs could therefore be on average greater or smaller than RFs in the same bat (Figure 5). Nevertheless, when comparing all bats, the values for RFs and SCF frequencies were positively correlated with one another (Figures 2,3), i.e. the higher the average RF was in any given bat the higher was also its average SCF (Linear regression, *R^2^* = 0.983, *F* = 349.384, *P* = 0.0000). Despite the fact that RFs and SCFs differed significantly from one another by amounts that changed from day to day, the average RFs and SCFs for each bat differed by usually no more than 1% of its SCF value (Table 2 and Figure 5).

**Figure 6:**
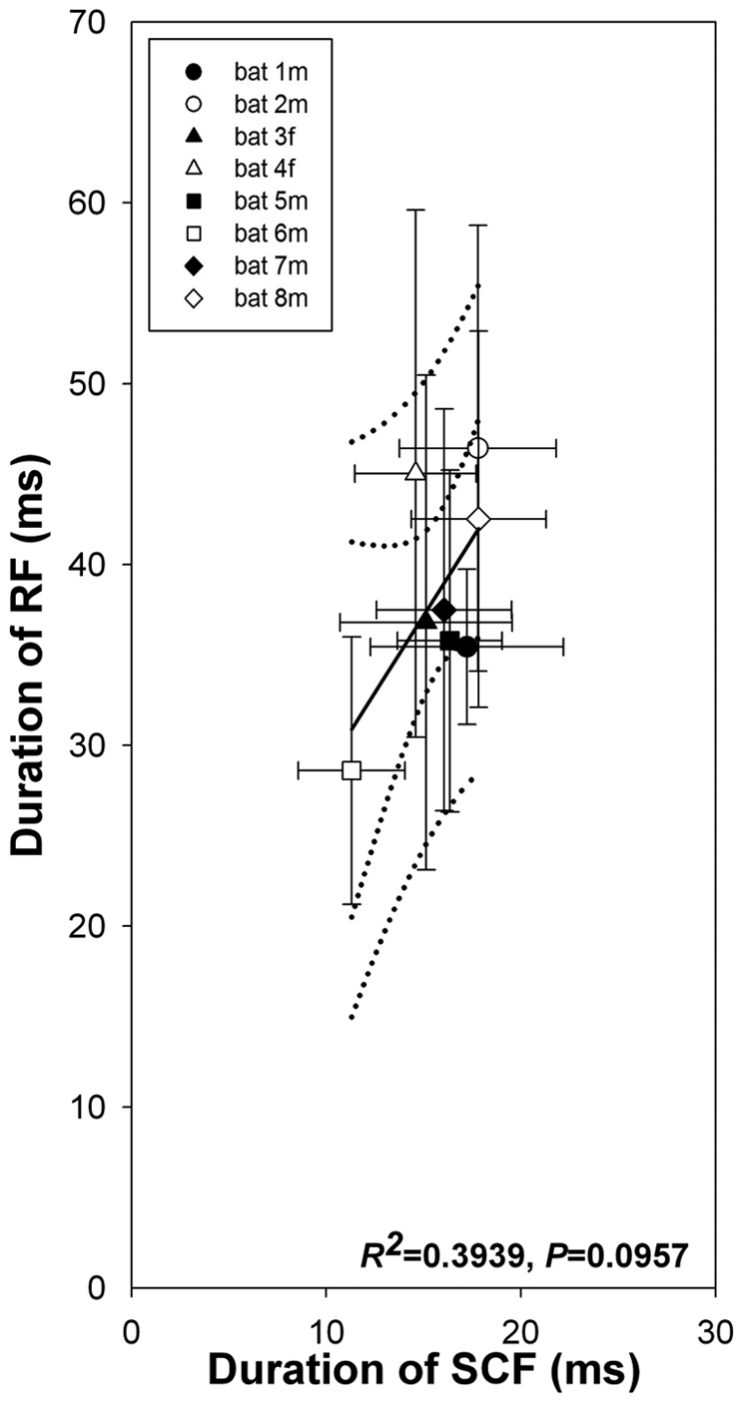
Distribution of the durations of RF and SCF calls for all 8 bats averaged over the entire observation period and presented as mean ± SD. Same data as in Table 2 (right half) with durations for RF calls emitted before and after a SCF sequence being averaged. Solid black line indicates the regression line, dotted innermost lines give the 95% confidence intervals, and dotted outermost lines outline the predicted intervals.

In contrast to the frequency values of RF and SCF calls, their durations did not differ significantly (Table 2 and Figure 6), although there was also a slight positive correlation between them, with shorter SCFs correlated with shorter RFs (Nonlinear regression, *R^2^* = 0.3939, *F* = 3.899, *P* = 0.0957). Finally, the CVs for the durations of RF and SCF calls (Fig. 4b) were more similar and did not exhibit the rather large difference seen for their frequencies (Fig. 4a; independent samples, T-test, *t* = −1.296, *P* = 0.216). CVs for RF call durations varied between 0.12 and 0.37 (mean ± SD: 0.268±0.0725) and those for SCFs between 0.19 and 0.29 (mean ± SD: 0.229±0.0435). It is noteworthy to mention that the durations of SCF calls are already short and yet the CVs of SCF calls vary more than those for their frequencies.

## Discussion

We found that the RFs of echolocation calls varied from day to day, sometimes by as much as 900 Hz, whereas SCF frequencies remained remarkably stable (Table 2, Figure 2). As a result, RFs for most individuals overlapped but SCF frequencies did not (Fig. 3; Tables S1, S2). In contrast, the durations of both call types, RF and SCF calls, lacked this large difference in variability (Table 2 and Figure 6).

Changes in the CF-frequency of echolocation pulses had been reported earlier and in several CF-species: In the Taiwanese leaf-nosed bat, RFs changed by an average of 3% during an observation period of several months with an average of 160 Hz/day, but in some instances could exceed 1 kHz/day [35]. In the mustache bat, changes in body temperature were correlated with changes in RFs of about 100 Hz/°C. These changes in RFs occurred concomitantly with changes in the fine tuning of the cochlea [36] and of auditory neurons within the CNS [37]. The results show that cochlear tuning in mustache bats is labile, and suggest shifts in the frequency-to-place code within those regions of the cochlea that are sharply tuned to the RFs, i.e. the auditory fovea. Hence the changes in RFs accommodate changes in the tuning of the auditory fovea, thus optimizing echolocation performance in response to changes in the auditory feedback [37]. The similarities in the echolocation systems of horseshoe and mustache bats [29], [30] suggest that the changes in echolocation call frequencies that we observed in horseshoe bats most likely also reflect a shift in the auditory fovea and thus changes in the auditory feedback provided by the cochlea.

Although the detailed time courses of the daily alterations in echolocation call frequencies have not been analyzed yet, it appears that they perhaps involve daily changes in the metabolic state of the animals, such as wake-sleep cycles. This is also corroborated by observations in *Hipposideros taiwanensis* and the mustache bat [35]–[37].

The spectral fine structure of communication calls also seems to depend on auditory feedback. Evidence for the role of auditory feedback in the acquisition and maintenance of individually distinct communication calls is based on studies in various species of bats (e.g., review by [2]). Playback experiments in pups of lesser spear-nosed bats, *Phyllostomus discolor*, demonstrated that they adjust their calls to playbacks of maternal directive calls [16]. Similarly, multivariate statistical analysis as well as playback experiments in adult greater spear-nosed bats, *Phyllostomus hastatus*, showed that a particular type of social call that is used to coordinate foraging flights between members of a certain social group were acquired through vocal learning [15]. Recent evidence for the importance of auditory feedback for social call structure has been provided by studies on the development of the territorial song in pups of the greater sac-winged bat, *Saccopteryx bilineata* [17]. The authors played back previously recorded territorial songs of different males to pups of different ages and find that the pups learn these complex vocalizations through vocal imitation by imitating the territorial song they were exposed to during development. The resemblance of pup vocalizations to their acoustic model is not caused by physical maturation effects. It also does not depend on the pups' gender and relatedness to adult males, and it becomes more pronounced during ontogeny. This clearly demonstrates the essential role of auditory experience for the development and maintenance of communication calls [17]. There is also evidence, however, that some communication signals in bats do not necessarily require auditory feedback and are perhaps not acquired through vocal learning. Pup isolation calls in the evening bat, *Nycticeius humeralis*, and vocal interactions during mother-offspring interactions in big brown bats, *Eptesicus fuscus*, for example may largely have a genetic basis ([5], [48], [49]; review: [2]).

The fact that the SCF frequencies remained remarkably stable may suggest that they allow for recognition of individuals, thus carrying an “individual frequency signature”. Vocal signatures have been demonstrated in mother-infant communications in other bats ([8], [50]–[52]; reviews: [53], [54]). More recently, it was shown that the isolation calls of noctule bat pups, *Nyctalus noctula*, were individually distinct and likely to bear vocal signatures suitable for acoustic mother-offspring recognition [55]. Noctule bats are one of the few species that usually give birth to twins. When comparing the variation of isolation calls of individuals within twin pairs with those between individuals from different twin pairs, they show that isolation calls of twin siblings were more similar to the calls of each other than to the isolation calls of unrelated pups of the same age. They concluded that isolation calls may therefore not only signal individual identity but also affiliation to a certain social group (e.g. twin pairs; [55]). Various studies tested the significance of vocal signatures for mother-offspring recognition through playback experiments, in which previously recorded isolation calls from different pups were broadcast to their respective mothers [5], [56], [57].

Assuming that SCF frequencies depend on auditory feedback, their high degree of constancy would suggest that cochlear input also remains constant, meaning that the frequency of the auditory feedback would not change. This in turn would then require different feedback loops from the cochlea for the control of RFs and SCFs, respectively: the location on the cochlear frequency map from which the feedback for RFs originates shifts back and forth on the basilar membrane together with the shifting auditory fovea whereas the location giving rise to the feedback for SCFs remains constant. It is unclear which mechanism enables auditory feedback to remain unaffected by the changes in the cochlear tuning and ultimately allow SCFs to be so remarkably stable. Alternatively, SCFs could also be completely independent of auditory feedback. Either scenario, however, signifies a fundamentally different mechanism underlying the frequency control of echolocation and communication calls.

One may argue that our observation period of less than one week is somewhat short to determine how auditory feedback may differ for RFs and SCFs. However, auditory feedback controls RFs on a call-by-call basis (e.g., [22], [31], [58]–[60]). Hence, assuming auditory feedback also controls social calls, such as SCFs, it is plausible that this also applies to them. Therefore our conclusion that auditory feedback differs for RF and SCF calls is not affected by the length of the observation period. Any day-to-day change in call frequency that we observed for RF calls does indeed require changes in the underlying auditory feedback. Conversely, any lack in call frequency changes, such as for SCFs, is consistent with no change in auditory feedback, or a complete lack thereof.

Our finding that the control of horseshoe bat echolocation and communication sounds involves different mechanisms of auditory feedback control is corroborated by neurobiological data indicating that the motor pathways for echolocation and communication calls are at least partially separate within the brainstem (review: [18]). Moreover, in mammals in general, different types of vocalization appear to involve different subsystems of the brainstem vocal motor network [19]. Although our data indicate that the auditory feedback control for different types of utterances differs as well, the neurobiological basis for this difference still remains to be elucidated.

## Materials and Methods

### Ethics Statement

All animal work has been conducted according to relevant national and international guidelines. All husbandry and experimental procedures were in accordance with NIH guidelines for experiments involving vertebrate animals and were approved by the Chancellor's Animal Research Committee of the University of California, Los Angeles (ARC 2001-108-22).

A total of 29 greater horseshoe bats, *Rhinolophus ferrumequinum tragatus*, from Northern China were housed in the bat facility at UCLA. Details of the animal husbandry have been described elsewhere [11] and are only summarized briefly here. The animal room had regulated light/dark cycles adjusted with an astronomical light timer to the natural photoperiod for North China (but time-inverted by approximately 12 hrs relative to the light-dark cycle at the study location at UCLA). The rooms were temperature and humidity controlled, with temperatures ranging between 25 and 30°C (40–60% rel. humidity) during the light period, and between 15 and 20°C during the dark period (50–80% rel. humidity). The colony was of mixed sex with approximately a 1∶1 ratio of males to females.

Calls were recorded between January and September, 2008. For each recording session, we separated 2 randomly chosen individuals (2 males, 2 females, or 1 male + 1 female) from the rest of the colony by transferring them into an observation cage (wire mesh cage; dimensions 0.6×0.6×1 m) that was positioned in the center of one room of the animal facility that did not contain any other bats. The 2 individuals were kept in the observation cage for up to 12 h with free access to food and water. The bats had distinct toe markings made with nail polish that allowed us to identify the bats individually. Of the 29 bats housed in the colony, the most vocally active bats (a total of 13 males and 7 females) were used in various combinations. All of these 20 bats produced echolocation pulses but only 6 males and 2 females produced SCFs. Thus, this study focuses on the analysis of these 8 bats. Only calls emitted at rest were recorded (RF calls, no DSC). Recordings were performed during the same time of day when the bats were vocally the most active, starting immediately after the lights turned off and lasting up to 8 hrs.

We used a commercially available ultrasonic acquisition system (UltraSoundGate 116, Avisoft Bioacoustics, Berlin, Germany; sample rate: 750 kHz, 8bit resolution) and sound analysis system (Avisoft-SASLab Pro, version 4.3; Avisoft Bioacoustics, Berlin, Germany). Sounds were stored and analyzed on computer using a sample rate of 250 kHz at 16 bits/sample. Bats were also observed under infrared illumination (wavelength 850 nm) using an infrared closed-circuit camera system (Q-See QS2814C, Digital Peripheral Solutions, Anaheim, CA). Video recordings of the bats' behavior and sound recordings were recorded and stored simultaneously on computer for subsequent analysis. This allowed us to relate the calls to certain behaviors, such as mating.

We obtained the call durations of RF and SCF calls from their waveforms (250 kHz sample rate). Spectrograms were only used for graphical presentation of the calls, such as in Figure 1, and were obtained using a 512 pt FFT (Hamming window) at a temporal resolution of 1.024 ms (frequency resolution: 244 Hz). The dominant frequency values of the constant frequency portions of RFs and SCFs were determined from the power spectra of individual calls (frequency resolutions between 5 and 10 Hz). Statistical analysis of the data was performed using commercial statistics software (SPSS, Chicago, IL, USA).

## Supporting Information

Table S1 **Multiple comparison among RFs of individuals in the center cluster of** **Figures 2** **,** **3** **(ANOVA, significance level: 0.05).**
(DOCX)

Table S2 **Multiple comparison among RFs of individuals in the left cluster of** **Figures 2** **,** **3** **(ANOVA, significance level: 0.05).**
(DOCX)

## References

[1] Doupe AJ, Kuhl PK (1999) Birdsong and human speech: Common themes and mechanisms. Ann Rev Neurosci 22: 567–631. 10202549 10.1146/annurev.neuro.22.1.567

[2] Boughman J, Moss C (2003) Social sounds: vocal learning and development of mammal and bird calls. In: Simmons A, Popper AN, Fay RR, eds. Acoustic communication., Springer handbook of auditory research. Berlin: Springer Press. 138–213.

[3] Janik VM, Slater PJB (1997) Vocal learning in mammals. In: Slater PJB, Rosenblatt JS, Snowdon CT, Milinski M, eds. Advances in the Study of Behavior, Vol. 26. San Diego and London: Academic Press, Inc. 59–99.

[4] Aldridge HDJN, Obrist M, Merriam HG, Fenton MB (1990) Roosting vocalizations and foraging by the African bat *Nycteris thebaica* . J Mamm 71: 242–246.

[5] Balcombe JP (1990) Vocal recognition of pups by mother Mexican free-tailed bats *Tadarida brasiliensis mexicana* . Anim Behav 39: 960–966.

[6] Behr O, Helversen Ov (2004) Bat serenades – complex courtship songs of the sac-winged bat (*Saccopteryx bilineata*). Behav Ecol Sociobiol 56: 106–115.

[7] Bohn KM, Schmidt-French B, Schwartz C, Smotherman M, Pollak GD (2009) Versatility and stereotypy of free-tailed bat songs. PloS One 4: e6746. 19707550 10.1371/journal.pone.0006746 PMC2727915

[8] Brown P (1976) Vocal communication in the Pallid Bat, *Antrozous pallidus* . Z Tierpsychol 41: 34–54. 961121 10.1111/j.1439-0310.1976.tb00469.x

[9] Fenton MB (1985) Communication in the Chiroptera. Bloomington: Indiana University Press. 161 p.

[10] Kanwal JS, Matsumura S, Ohlemiller K, Suga N (1994) Analysis of acoustic elements and syntax in communication sounds emitted by mustached bats. J Acoust Soc Am 96: 1229–1254. 7962992 10.1121/1.410273

[11] Ma J, Kobayasi K, Zhang S, Metzner W (2006) Vocal communication in adult greater horseshoe bats, *Rhinolophus ferrumequinum* . J Comp Physiol A 192: 535–550. 10.1007/s00359-006-0094-9 16418857

[12] Matsumura S (1981) Mother-infant communication in a horseshoe bat (*Rhinolophus ferrumequinum nippon*): vocal communication in three-week-old infants. J Mammol 62: 20–28.

[13] Pfalzer G, Kusch J (2003) Structure and variability of bat social calls: implications for specificity and individual recognition. J Zool Lond 261: 21–33.

[14] Russo D, Jones G (1999) The social calls of Kuhl's pipistrelles *Pipistrellus kuhlii* (Kuhl, 1819): Structure and variation (Chiroptera: Vespertilionidae). J Zool Lond 249: 476–481.

[15] Boughman JW (1998) Vocal learning by greater spear-nosed bats. Proc R Soc Lond B Biol Sci 265: 227–233. 10.1098/rspb.1998.0286 PMC1688873 9493408

[16] Esser KH (1994) Audio-vocal learning in a non-human mammal: the lesser spear-nosed bat, *Phyllostomus discolor* . Neuroreport 5: 1718–1720. 7827315 10.1097/00001756-199409080-00007

[17] Knörnschild M, Nagy M, Metz M, Mayer F, von Helversen O (2010) Complex vocal imitation during ontogeny in a bat. Biol Lett 6: 156–159. 19812069 10.1098/rsbl.2009.0685 PMC2865031

[18] Fenzl T, Schuller G (2007) Dissimilarities in the vocal control over communication and echolocation calls in bats. Behav Brain Res 182: 173–179. 17227683 10.1016/j.bbr.2006.12.021

[19] Hage SR (2010) Neuronal networks involved in the generation of vocalization. In: Brudzynski SM, eds. Handbook of mammalian vocalization. New York: Academic Press. 339–349.

[20] Möhres FP (1953) Über die Ultraschallorientierung der Hufeisennasen (Chiroptera-Rhinolophinae). Z Vergl Physiol 34: 547–588.

[21] Neuweiler G, Metzner W, Heilmann U, Rübsamen R, Eckrich M, et al (1987) Foraging behaviour and echolocation in the rufous horseshoe bats, *Rhinolophus rouxi*, of Sri Lanka. Behav Ecol Sociobiol 20: 53–67.

[22] Schnitzler HU (1968) Die Ultraschallortungslaute der Hufeisennasen-Fledermäuse (Chiroptera, Rhinolophidae) in verschiedenen Orientierungssituationen. Z Vergl Physiol 57: 376–408.

[23] Jones G, Rayner JMV (1989) Foraging Behavior and Echolocation of Wild Horseshoe Bats *Rhinolophus ferrumequinum* and *R. hipposideros* (Chiroptera, Rhinolophidae). Behav Ecol Sociobiol 25: 183–191.

[24] Schnitzler HU, Denzinger A (2011) Auditory fovea and Doppler shift compensation: adaptations for flutter detection in echolocating bats using CF-FM signals. J Comp Phsyiol A 197: 541–559. 10.1007/s00359-010-0569-6 20857119

[25] Altes R (1976) Sonar for generalized target description and its similarity to animal echolcoation systems. J Acoust Soc Am 59: 97–105. 1249325 10.1121/1.380831

[26] Goldman L, Henson O (1977) Prey recognition and selection by the constant frequency bat, *Pteronotus parnellii parnellii* . Behav Ecol Sociobiol 2: 411–420.

[27] Neuweiler G (1990) Auditory adaptations for prey capture in echolocating bats. Physiol Rev 70: 615–641. 2194220 10.1152/physrev.1990.70.3.615

[28] Schuller G, Pollak GD (1979) Disproportionate frequency representation in the inferior colliculus of Doppler-compensating Greater Horseshoe Bats, *Rhinolophus ferrumequinum* . J Comp Physiol 132: 47–54.

[29] Vater M, Kössl M (2004) The ears of whales and bats. In: Thomas J, Moss C, Vater M, eds. Echolocation in bats and dolphins. Chicago: Univ Chicago Press. 89–99.

[30] Kössl M (1994) Evidence for a mechanical filter in the cochlea of the ‘constant frequency’ bats, *Rhinolophus rouxi* and *Pteronotus parnellii* . Hear Res 72: 73–80. 8150747 10.1016/0378-5955(94)90207-0

[31] Schuller G, Beuter K, Schnitzler HU (1974) Response to frequency-shifted artificial echoes in the bat, *Rhinolophus ferrumequinum* . J Comp Physiol 89: 275–286.

[32] Metzner W, Zhang SY, Smotherman MS (2002) Doppler-shift compensation behavior in horseshoe bats revisited: auditory feedback controls both a decrease and an increase in call frequency. J Exp Biol 205: 1607–1616. 12000805 10.1242/jeb.205.11.1607

[33] Grinnell AD (1989) Sensory-motor control: listening to the voice within. Nature 341: 488–489. 2797176 10.1038/341488a0

[34] Ruebsamen R, Schaefer M (1990) Audiovocal interactions during development? Vocalisation in deafened young horseshoe bats vs. audition in vocalisation-impaired bats. J Comp Physiol A 167: 771–784. 2086791 10.1007/BF00189767

[35] Hiryu S, Kastsura K, Nagato T, Yamazaki H, Lin LK, et al (2006) Intra-individual variation in the vocalized frequency of the Taiwanese leaf-nosed bat, *Hipposideros terasensis*, influenced by conspecific colony members. J Comp Physiol A 192: 807–815. 10.1007/s00359-006-0118-5 16538514

[36] Huffman RF, Henson OW (1993) Labile Cochlear Tuning in the Mustached Bat I. Concomitant Shifts in Biosonar Emission Frequency. J Comp Physiol A 171: 725–734. 8441121 10.1007/BF00213069

[37] Huffman RF, Henson OW (1993) Labile Cochlear Tuning in the Mustached Bat II. Concomitant Shifts in Neural Tuning. J Comp Physiol A 171: 735–748. 8441122 10.1007/BF00213070

[38] Jones G, Ransome RD (1993) Echolocation calls of bats are influenced by maternal effects and change over a lifetime. Proc R Soc Lond B Biol Sci 252: 125–128. 10.1098/rspb.1993.0055 8391702

[39] Suga N, Niwa H, Taniguchi I, Margoliash D (1987) The personalized auditory cortex of the mustached bat: adaptation for echolocation. J Neurophysiol 58: 643–654. 3681389 10.1152/jn.1987.58.4.643

[40] Fenton MB (1977) Variations in the social calls of little brown bats (*Myotis lucifugus*). Can J Zool 55: 1151–1157.

[41] Boughman JW, Wilkinson GS (1998) Greater spear-nosed bats discriminate group mates by vocalizations. Anim Behav 55: 1717–1732. 9642014 10.1006/anbe.1997.0721

[42] Wilkinson GS, Wenrick-Boughman J (1998) Social calls coordinate foraging in greater spear-nosed bats. Anim Behav 55: 337–350. 9480702 10.1006/anbe.1997.0557

[43] Bohn KM, Schmidt-French B, Ma ST, Pollak GD (2008) Syllable acoustics, temporal patterns, and call composition vary with behavioral context in Mexican free-tailed bats. J Acoust Soc Am 124: 1838–1848. 19045674 10.1121/1.2953314 PMC2676615

[44] Fenton MB (1994) Communication in Chiroptera. Bloomington: Indiana University Press.

[45] Jones G, Sripathi K, Waters DA, Marimuthu G (1994) Individual variation in the echolocation calls of three sympatric Indian hipposiderid bats, and an experimental attempt to jam bat echolocation. Folia Zool 43: 347–362.

[46] Masters WM, Raver KAS, Kazial KA (1995) Sonar signals of big brown bats, *Eptesicus fuscus*, contain information about individual identity, age and family affiliation. Anim Behav 50: 1243–1260.

[47] Tian B, Schnitzler HU (1997) Echolocation signals of the greater horseshoe bat (*Rhinolophus ferrumequinum*) in transfer flight and during landing. J Acoust Soc Am 101: 2347–2364. 9104033 10.1121/1.418272

[48] Masters WM, Raver KAS, Kazial KA (1995) Sonar signals of big brown bats, *Eptesicus fuscus*, contain information about individual identity, age and family affiliation. Anim Behav 50: 1243–1260.

[49] Scherrer J, Wilkinson G (1993) Evening bat isolation calls provide evidence for heritable signatures. Anim Behav 46: 847–860.

[50] Barclay RMR, Thomas DW (1979) Copulation call of *Myotis lucifugus*: a discrete situation specific communication signal. J Mammal 60: 632–634.

[51] Jones G, Hughes PM, Rayner JMV (1991) The development of vocalizations in *Pipistrellus pipistrellus* (Chiroptera, Vespertilionidae) during post-natal growth and the maintenance of individual vocal signatures. J Zool Lond 225: 71–84.

[52] Schmidt U, Joerman G, Schmidt C (1981) Struktur und Variabilität der Verlassenheitslaute juveniler Vampirfledermäuse (*Desmodus rotundus*). Zeitschr Säugetierk 47: 143–149.

[53] Kunz TH, Hood W (2000) Parental care and postnatal growth in the chiroptera. In: Crichton E, Krutzsch P, eds. Reproductive biology of bats. London: Academic Press. 415–468.

[54] Wilkinson PR (2003) Social and vocal complexity in bats. In: Frans B, Tyack P, eds. Animal social complexity: Intelligence, culture, and individualized societies. Cambridge MA: Harvard Univ Press. 322–341.

[55] Knörnschild M, Helversen O, Mayer F (2007) Twin siblings sound alike: isolation call variation in the noctule bat, *Nyctalus noctula* . Anim Behav 74: 1055–1063.

[56] Thomson CE, Fenton MB, Barclay RMR (1985) The role of infant isolation calls in mother-infant reunions in the little brown bat *Myotis lucifugus* . Can J Zool 63: 1982–1988.

[57] De Fanis E, Jones G (1996) Allomaternal care and recognition between mothers and young in pipistrelle bats (*Pipistrellus pipistrellus*). J Zool Lond 240: 781–787.

[58] Schuller G (1986) Influence of echolocation pulse rate on Doppler-shift compensation control system in the Greater Horseshoe Bat. J Comp Physiol A 158: 239–246.

[59] Smotherman M, Metzner W (2003) Fine control of call frequency by horseshoe bats. J Comp Physiol A 189: 435–446. 10.1007/s00359-003-0422-2 12761645

[60] Smotherman M, Metzner W (2005) Auditory-feedback control of temporal call patterns in echolocating horseshoe bats. J Neurophysiol 93: 1295–1303. 15496485 10.1152/jn.00653.2004

