# Supplementary material for: Different Auditory Feedback Control for Echolocation and Communication in Horseshoe Bats
Source: PLoS One. 2013 Apr 24;8(4):e62710. doi: 10.1371/journal.pone.0062710 (PMC3634746; doi:10.1371/journal.pone.0062710)
Supplement: Table S1 — Multiple comparison among RFs of individuals in the center cluster of Figures 2 , 3 (ANOVA, significance level: 0.05). (DOCX) [file pone.0062710.s001.docx]

Table S1: Multiple comparison among RFs of individuals in the center cluster of Figures 2,3 (ANOVA, significance level: 0.05).

| Individual | Individual | *P* | *Std.Error* |
| --- | --- | --- | --- |
| 3f | 4f | 0.000 | 14.26996 |
|  | 5m | 0.000 | 8.17496 |
| 4f | 3f | 0.000 | 14.26996 |
|  | 5m | 0.114 | 14.23971 |
| 5m | 3f | 0.000 | 8.17496 |
|  | 4f | 0.114 | 14.23971 |
