# Supplementary material for: Different Auditory Feedback Control for Echolocation and Communication in Horseshoe Bats
Source: PLoS One. 2013 Apr 24;8(4):e62710. doi: 10.1371/journal.pone.0062710 (PMC3634746; doi:10.1371/journal.pone.0062710)
Supplement: Table S2 — Multiple comparison among RFs of individuals in the left cluster of Figures 2 , 3 (ANOVA, significance level: 0.05). (DOCX) [file pone.0062710.s002.docx]

Table S2: Multiple comparison among RFs of individuals in the left cluster of Figures 2,3 (ANOVA, significance level: 0.05).

| Individual | Individual | *P* | *Std.Error* |
| --- | --- | --- | --- |
| 6m | 7m | 0.000 | 15.43466 |
|  | 8m | 0.000 | 12.20452 |
| 7m | 6m | 0.000 | 15.43466 |
|  | 8m | 0.439 | 12.38285 |
| 8m | 6m | 0.000 | 11.28289 |
|  | 7m | 0.439 | 12.38285 |
